# What Happened to Gray Whales during the Pleistocene? The Ecological Impact of Sea-Level Change on Benthic Feeding Areas in the North Pacific Ocean

**DOI:** 10.1371/journal.pone.0021295

**Published:** 2011-07-06

**Authors:** Nicholas D. Pyenson, David R. Lindberg

**Affiliations:** 1 Department of Paleobiology, National Museum of Natural History, Smithsonian Institution, Washington, D.C., United States of America; 2 Departments of Mammalogy and Paleontology, Burke Museum of Natural History and Culture, University of Washington, Seattle, Washington, United States of America; 3 Department of Integrative Biology, Center for Computational Biology and Museum of Paleontology, University of California, Berkeley, California, United States of America; University College London, United Kingdom

## Abstract

**Background:**

Gray whales (*Eschrichtius robustus*) undertake long migrations, from Baja California to Alaska, to feed on seasonally productive benthos of the Bering and Chukchi seas. The invertebrates that form their primary prey are restricted to shallow water environments, but global sea-level changes during the Pleistocene eliminated or reduced this critical habitat multiple times. Because the fossil record of gray whales is coincident with the onset of Northern Hemisphere glaciation, gray whales survived these massive changes to their feeding habitat, but it is unclear how.

**Methodology/Principal Findings:**

We reconstructed gray whale carrying capacity fluctuations during the past 120,000 years by quantifying gray whale feeding habitat availability using bathymetric data for the North Pacific Ocean, constrained by their maximum diving depth. We calculated carrying capacity based on modern estimates of metabolic demand, prey availability, and feeding duration; we also constrained our estimates to reflect current population size and account for glaciated and non-glaciated areas in the North Pacific. Our results show that key feeding areas eliminated by sea-level lowstands were not replaced by commensurate areas. Our reconstructions show that such reductions affected carrying capacity, and harmonic means of these fluctuations do not differ dramatically from genetic estimates of carrying capacity.

**Conclusions/Significance:**

Assuming current carrying capacity estimates, Pleistocene glacial maxima may have created multiple, weak genetic bottlenecks, although the current temporal resolution of genetic datasets does not test for such signals. Our results do not, however, falsify molecular estimates of pre-whaling population size because those abundances would have been sufficient to survive the loss of major benthic feeding areas (i.e., the majority of the Bering Shelf) during glacial maxima. We propose that gray whales survived the disappearance of their primary feeding ground by employing generalist filter-feeding modes, similar to the resident gray whales found between northern Washington State and Vancouver Island.

## Introduction

Between 50,000 to 10,000 years ago (50–10 ka), entire assemblages of large terrestrial mammal species perished on different continents throughout the world [1], [2]. The causes of the late Pleistocene megafaunal extinction have been hotly debated over the past 30 years, with mechanisms ascribed to both human agency as well as climatic perturbations [3], [4]. In contrast to terrestrial mammals, marine mammals appear to have survived late Pleistocene effects of climate change, with select species extinctions entirely attributable to human hunting and human-mediated habitat deterioration [5]–[8]. It has been argued that the protracted survival of marine mammals through to the Holocene occurred because of a comparatively longer delay in human capacity to extirpate marine mammals, whose ecology presented logistical and technological challenges for hunting [7], [9]–[11]. In the past few centuries, human impacts on marine mammals have accelerated in rate and expanded in scope, owing to both technological innovations and human practices (e.g., industrial whaling, fisheries by-catch), which have seriously threatened many populations and species with extinction [12], [13]. Recent analyses of molecular data suggest that some of the most heavily hunted marine mammals (i.e., large cetaceans) were several magnitudes more abundant prior to large-scale hunting [14], [15], calling into question the baselines used in modern-day marine mammal conservation and management debates [15], [16].

Gray whales (*Eschrichtius robustus*) are perhaps the most prominent example of successful conservation practices, which restored their population from several thousand individuals after whaling to more than 20,000 (20 k) individuals today. Gray whales ranged throughout the North Atlantic Ocean as recently as the 17^th^ century [17]–[19], but today they are limited to two populations in the North Pacific Ocean: western gray whales, consisting of a highly threatened population numbering in the hundreds that are thought to migrate between the coast of China and the Sea of Okhotsk [20]–[22]; and a comparatively larger population of eastern or California gray whales [23]–[24]. The natural history of the eastern gray whales has been studied since the mid-nineteenth century [25], and they are characterized by several features, including their annual migration from Baja California to Alaska, their nearshore habits and their ability to feed on benthic invertebrates using a modified mode of suction feeding, in addition to a generalized mode of filter feeding in the water column [26], [27].

The same features that made gray whales among the best-known mysticete species in the world (including their proximity to major centers of research on the west coast of North America) were also features that made them prime targets for shore- and ship-based whaling through the 19^th^ century [28], [29]. An international moratorium in the early 20th century allowed gray whale populations to rebound from near collapse, and subsequent legal protection by the U.S. Marine Mammal Act in 1972 continues to protect the eastern Pacific population [30]. Current assessments of the eastern gray whale population size, using shore- and sea-based surveys, indicate that this population has recovered to one estimate of pre-whaling population size (∼15 k to 20 k individuals [31]–[33]). This census-based estimate, however, conflicts by orders of magnitude with molecular analyses of gray whale genetic diversity [15], which suggest that pre-whaling population sizes were dramatically larger, up to 118 k individuals. (This molecular estimate covered the entirety of possible North Pacific metapopulations, although distributing the mean molecular value across both western and eastern population still indicates that eastern gray whales are 28–56% of their historical abundances [15]). If the molecular data on historical, pre-whaling estimates of gray whale population size are accurate, then the fixation of today's population at much lower carrying capacity may indicate that the structure of nearshore ecosystems in the North Pacific has fundamentally changed over the past few centuries [15], [31]. Such an argument fits into a broader set of evidence from changing marine mammal population distributions, sea ice reduction and drop in benthic invertebrate community biomass that coincide with oceanographic and climatic shifts, pointing to large-scale changes in the ecosystem function in the Bering Sea over the past two decades [34].

The main feeding habitat for the majority of gray whales is the shallow, benthic habitats on the shelf of the Bering Sea [26], [35]–[37]. The Bering Sea itself is one of the most productive marine ecosystems in the world [34], and its seasonally abundant resources provide the primary food for many species of large pelagic and nearshore vertebrates [38], [39]. This region has also been subject to geological influences during the past ∼0.5 Ma [40], [41], including changes in uplift, subsidence, rates of sedimentation, and global sea-level, concomitant with episodes of glaciation, which also modified ocean currents and circulation in the Bering Sea [42].

**Figure 1:**
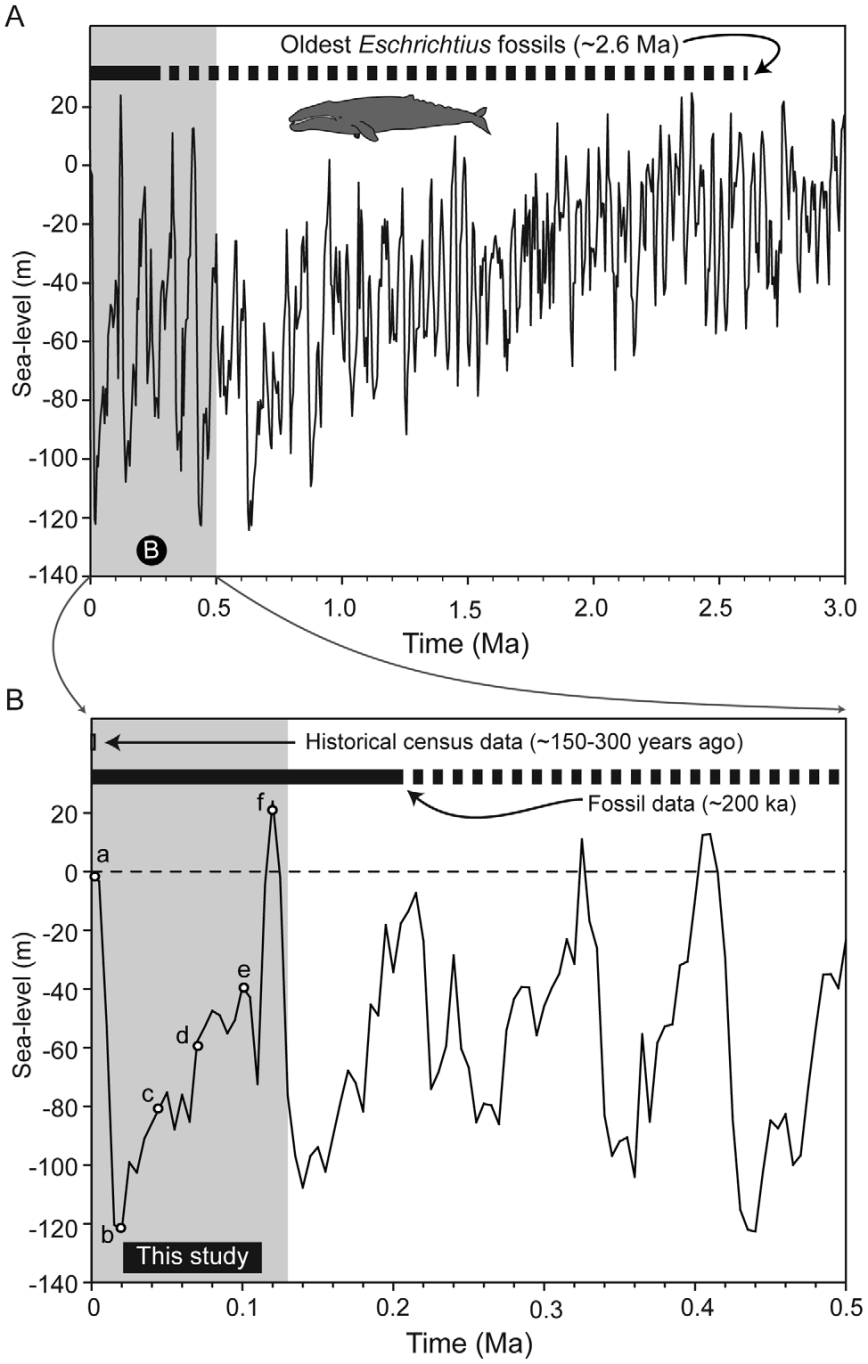
Pliocene through Holocene eustatic sea-level changes, at two different scales. Sea-level change [43] juxtaposed with A) the oldest known fossil belonging to the genus *Eschrichtius*, from the Pliocene of Japan (dashed line) [98]; and B) the relative temporal ranges from other historical gray whale data, with the oldest example belonging to the species occurrence (solid line), from the Palos Verdes Peninsula of California [101], [104]. Age for census estimates reflects an upper bound for reports from the written historical record [28], [29], [92], [93].

During Pleistocene glacial maxima, current shallow marine benthic environments that compose a large percentage of gray whale feeding grounds were eliminated by eustatic sea-level lows [35], [37]. These modifications to gray whale benthic feeding areas (and migration routes) occurred not just once, but numerous times during high-order (10^4^–10^5^ year) Pleistocene sea-level cycles [43]. The impact of these changes on marine mammal evolution is difficult to discern because their fossil record from this time is poorly sampled; moreover, much of the marginal marine rocks of Pleistocene age are inaccessible because they are located at depth several kilometers offshore from current coastlines [44]. Despite this bias, diagnostic gray whale fossils have been reported from both Pleistocene and late Pliocene marine strata of the North Pacific basin, attesting to the origin of this lineage prior to the onset of Northern Hemisphere glaciation in the late Pliocene (**Figure 1**).

Given this evidence, it is clear that gray whales have survived multiple glacial-interglacial periods, with concomitant changes to their critical feeding habitat. To investigate how gray whales survived the Pleistocene glaciations, we evaluated how the loss of feeding areas impacted their carrying capacity (a proxy for population size) by analyzing changes in benthic feeding area through time, as measured by available benthos from available bathymetry data [45]. We created a chronicle of benthic feeding area availability (and, by extension, carrying capacity estimates) by plotting bathymetry across a record of eustatic sea-level change during the past 120 ka within a window of their known feeding depth [46]. With estimations of benthic feeding area, we then calculated carrying capacity based on modern estimates of metabolic demand [15], prey availability [26], and feeding duration [47]. We also determined habitat availability relative to glaciated and non-glaciated areas of the continental margins along the North Pacific Ocean (**Figure S1**), and then recalculated carry capacity estimates that were constrained to reflect current population size [33].

We grounded our analysis in uniformitarian principles that extended known ecological parameters in a conservative fashion across a chronicle of sea-level changes through geologic time. This chronicle, along with attendant changes in benthic area along the western and eastern continental margins of the North Pacific Ocean, provided an overall line of evidence from Earth history to test hypotheses about the impact of large-scale habitat changes on the evolution of ecologically important consumers in nearshore communities, independent of the history of these consumers in their communities. Our results outline three major findings for the ecological history of gray whales: first, if census estimates of pre-whaling carrying capacity are correct, glacial episodes might have forced gray whales into low enough numbers to have caused multiple genetic bottlenecks; such signals, however, would be deeper temporally than any simulation of genetic diversity that has been conducted thus far. Second, fluctuations in benthos availability, over the past 120 ka, do not falsify molecular estimates of gray whale carrying capacity. Third, we suggest that gray whales survived Pleistocene glacial maxima and maintained substantial population sizes by employing a diverse set of feeding modes, similar to those seen in seasonal resident whales found today between northern Washington State and the coast of Vancouver Island [48].

## Results

**Figure 2:**
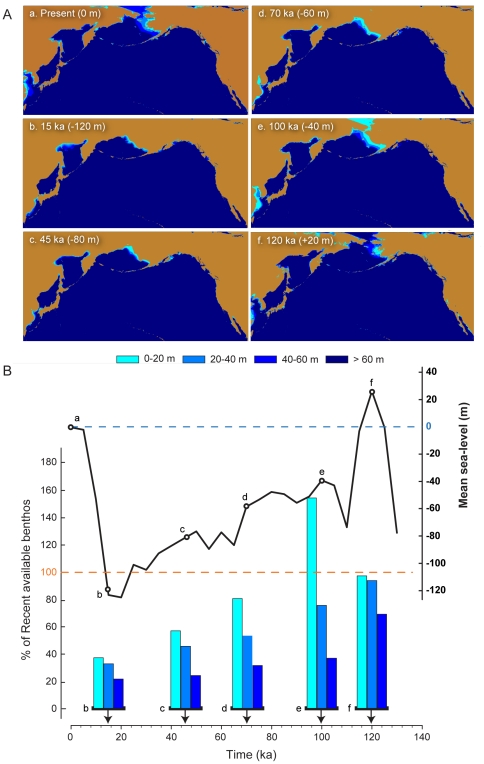
Benthos availability, sea-level change and coastal configuration of continental margins in the North Pacific Ocean, at select intervals, during the last 120 ka. A) Geographic plates at the top of the figure depict reconstructed coastal configurations and 20 m depth contours for (a) present day, (b) 15 ka, (c) 45 ka, (d) 70 ka, (e) 100 ka, and (f), 120 ka. See Table 1 for regional boundaries and summary data; depth data from ETOPO1 [115]. B) Left axis on the plot shows bar graphs with available benthos at 20 m increments at select time intervals (a–f). Right axis shows mean sea-level changes in past 130 ka, using data from Miller et al. [43]. Dashed lines indicate (left, in orange) current sea-level and (right, in blue) current benthos availability.

Our results showed that potential benthic feeding areas for North Pacific gray whale populations varied markedly in time and space during the last interglacial-glacial cycle (**Figure 2**), although several notable patterns emerged in our analysis. Overall, benthos availability has been historically lower than present, with 33% of the current level of benthos availability (within 75 m of the surface) during the last glacial maximum (LGM; **Table 1**). In our reconstructions, less than 60% of the modern North Pacific benthos area was available during two periods (15–65 ka) and at 110 ka, while only on two occasions did benthos availability equal or exceed the current level (5 and 120 kya) (**Table S1**).

**Table 1:** Regional definitions and current, minimum, and maximum benthos area available within 75 m of the surface during the last glacial cycle (120 ka), including the age and percent of current benthos of past minimum and maximum events.

					Recent Benthoskm^2^	Min Benthos		% ofRecent	Max Benthos		% ofRecent
Region	Lat°	Long°	Lat°	Long°		km^2^	ka		km^2^	ka	
East Pacific South (EPS)	49.00 N	109.90 W	22.83 N	126.00 W	56271	28445	20	51%	64771	105	115%
East Pacific North (EPN)	62.00 N	129.00 W	49.00 N	180.00 W	867193	216875	20	25%	895340	120	103%
West Pacific North (WPN)	60.00 N	180.00 W	50.00 N	130.00 E	226031	195096	85	86%	249383	55	110%
West Pacific South (WPS)	50.00 N	160.00 E	30.00 N	115.00 E	733964	160806	20	22%	733964	0	100%
North Pacific (NP)	all regions				1883459	618286	20	33%	1915662	120	101%

See Materials and Methods and Fig. 1 for data source and treatment.

**Figure 3:**
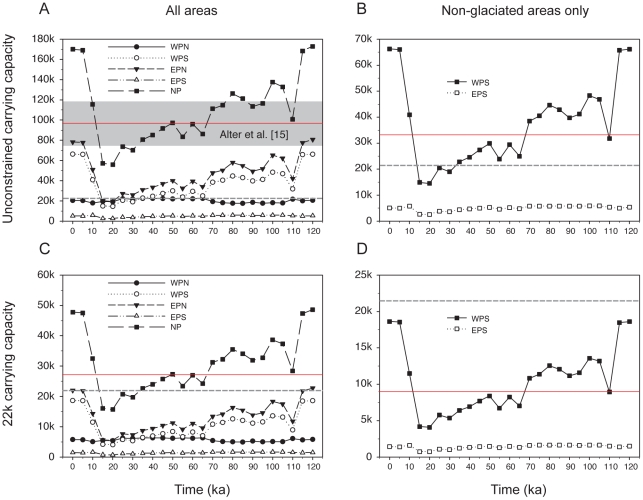
Estimated carrying capacity for North Pacific gray whales determined by benthos availability (<75 m) during the last 120 ka. Dashed gray lines indicate current gray whale population size and red lines show harmonic means for carrying capacity estimates. A) Total and regional North Pacific unconstrained carrying capacities. Gray box indicates range of population size suggested by analysis of genetic diversity [15]. B) Total and regional North Pacific carrying capacities constrained to 22 k gray whales; see Table 2; C) Unconstrained, estimated carrying capacities of non-glaciated regions in the western and eastern Pacific; D) Estimated carry capacities of non-glaciated regions in the western and eastern Pacific constrained to 22 k gray whales; see Table 2.

Fluctuations in eustatic sea-level changed the location and amount of available benthic food resources for gray whales during the late Pleistocene (**Figure 2**). Our analyses indicated that the gray whale carrying capacity in the North Pacific Ocean could have been as high 172,946 individuals (at 120 ka; **Figure 3A** **, Table S2**), assuming modern day values for benthic productivity, food density, and gray whale energetics. Of this estimated abundance, 85% of the individuals would have been potentially supported by the northeast and southwest Pacific regions, with 80,831 and 66,154 individuals, respectively (EPN and WPS in **Figure 3A**), while the northwest and southeast regions would have supported only 5,324 and 5,801 individuals, respectively; 15% of the estimated carrying capacity at 120 ka (WPN and EPS in **Figure 3A**). Although the northeastern Pacific region (Bering and Chukchi Seas, in the EPN region of **Figure 3A**) provided the highest amount of available benthos for gray whales, the southwestern Pacific region would have supported the most gray whales along the coast of Asia (WPS in **Figure 3A**). Most critically, the eastern Pacific regions south of the Bering shelf (southeast Alaska to Baja California) could not have compensated for the loss of benthic feeding area to the north (**Figure 3A, B**) because a commensurate amount of benthos within a −75 m window was not otherwise available during glacial maxima in these unglaciated regions.

The harmonic means (HM) of our estimated carrying capacities between 15–120 ka are presented as red lines in **Figure 3**. For an unconstrained estimate of carrying capacity in the entire North Pacific (**Figure 3A**), HM was 96,284 individuals, while limiting the population to only the non-glaciated areas lowered the HM to 34,308 individuals (**Figure 3B**). We also constrained the carrying capacity of the North Pacific population to 22 k individuals (**Figure 3C,D**), based on recent population surveys which have produced the census-based estimates of gray whale population size [33]. We calculated this value by requiring the modern EPS and EPN population sizes to sum to 22 k, and then scaled the ratio of food patch to area to support only 22 k individuals (**Table 2**).

**Table 2:** Parameters and sources used to estimate carrying capacities for gray whales in the North Pacific Ocean during the last 120 ka.

Assumptions	Prey biomass requirement	Feeding window	Prey density	Ratio food patch to area	Carrying Capacity
Data Source	Alter et al. [15]	Johnson and Nelson [47]	Nerini [26]	Nerini [26]	Rugh et al. [33]
Unconstrained	366 kg individual^−1^ day^−1^	180 days	161 g/m^2^	0.03722	—
Alternative Bering SeaCarrying Capacity	366 kg individual^−1^ day^−1^	180 days	161 g/m^2^	0.0104	22,000

To limit carrying capacity, the ratio of food patch size to area was reduced to support only 22 k gray whales based on the current eastern Pacific population size [33].

Constraining the carrying capacity to 22 k individuals lowered the HM for the North Pacific (**Figure 3C**) to 27,064 individuals and in the non-glaciated regions to 9,643 individuals (**Figure 3D**). By comparison, an average carrying capacity of ∼96 k individuals was calculated from genetic data by Alter et al. [15] for the entire North Pacific Ocean.

The carrying capacities in **Figure 3** assumed that gray whales were feeding up to the edge of pack and fast ice, as well as the margins of continental and island glaciers. In the eastern Pacific, however, non-glaciated regions were available only south of the Cordilleran glacial complex (i.e., Puget Sound, Washington State) (**Figure S1**). Thus, if we assumed that gray whales avoided glacial and pack ice conditions, and, for a time, were restricted to non-glaciated regions, available benthos and our results show that there was no interval of time during the last 120 ka when the estimated population size exceeded 6 k individuals in the unconstrained estimates (EPS in **Figure 3B**) or 1.7 k individuals in estimates constraining the carrying capacity to 22 k individuals (EPS in **Figure 3D**). In contrast, the non-glaciated regions in the western Pacific (WPS area south of the Kamchatka Peninsula, Russia; **Figure S1**) could have supported substantially more gray whales, and even during glacial maxima, the WPS non-glaciated regions would have had a minimum carrying capacity of 14.5 k individuals and approximately 4 k individuals with the carrying capacity constrained to 22 k individuals (WPS in **Figures 3B and 3D**, respectively). We suspect, however, that gray whale restriction to non-glaciated regions most likely occurred only during glacial maxima (i.e., in this study, LGM, ∼15–20 ka). Prior to this time, the buildup of continental and island glaciers between 20 ka and 115 ka more likely resulted in the gradual loss of potential feeding habitat as the glaciers expanded southward over 95 k years. Thus, the carrying capacity estimates for the glaciated regions would have fallen over time rather than been constantly unavailable, and the HM for carrying capacities would have been higher than the 27,064 individuals shown in **Figure 3C**, but also lower than 96,283 individuals given in **Figure 3A**.

## Discussion

As iconic exemplars of successful marine mammal conservation, the status of living gray whale populations in the North Pacific Ocean remains a topic of perennial concern. To better understand the range of their ecological variability for evaluating baseline conditions, researchers have attempted to reconstruct the deeper history of gray whales using different kinds of datasets. Recent census and survey efforts suggest that eastern North Pacific gray whales, which account for the vast majority of all living gray whales, have reached a stable size ∼22 k individuals, a value that largely agrees with pre-whaling estimates based on logbooks and anecdotal accounts from the 19^th^ century [31]–[33]. In contrast, Alter et al. [15] estimated a North Pacific population of between 76 k–118 k individuals based on genetic diversity, a range 2–3 times larger than uppermost estimates based on written historical and observational data. This discrepancy has led to an ongoing debate about the relative accuracy of genetic data for estimating past population sizes, and the impact of successive eras of whaling [17], [49].

Our analysis used uniformitarian assumptions about gray whale feeding ecology and reconstructed their carrying capacity using fundamental constraints from Earth history (i.e., benthos availability resulting from sea-level change), which we chronicled across geologic time. Most notably, our results do not falsify the large Holocene population sizes that Alter et al. [15] calculated; more than half of our unconstrained carrying capacity estimates, as well as the harmonic mean of our estimates for the entire North Pacific, fall within Alter et al. [15]'s range of carrying capacity estimates (**Figure 3A** **, Table S2**). Our results also suggest that substantial population growth would have been initiated around 15 ka (post-LGM), reaching a plateau around 5 ka.

If our estimates are constrained to a 22 k carrying capacity, then our results suggest that eastern North Pacific gray whale carrying capacity would have been reduced to less than 10 k individuals for over 20 ka prior to the recent growth (**Figure 3B**). In the western North Pacific, the 22 k carrying capacity constraint would mean that the ancestral population size of gray whales would have also been reduced to less than 10 k individuals, but only for 15 ka because of the greater carrying capacity of the WPS region (**Figure 3B** **, Table S1**).

### Uniformitarian assumptions and alternative hypotheses

Our analysis used uniformitarian assumptions about current values in gray whale feeding ecology to limit the range of possible geographic scenarios, concomitant with continental and pack ice extent and available benthos during sea-level changes in the last 120 ka. Although organismal traits such as geographic range, feeding duration, and diving depth and duration can vary and change over geologic time, we had no *a priori* rationale for selecting traits besides those known to us from primary references in the gray whale feeding literature. In a strict view, uniformitarianism is an argument for assuming constancy in static values as well as process-based ones (e.g., rates). Without any evidence to suggest otherwise, we have projected select modern day values of gray whale feeding ecology into the past. For example, one crucial value that we used was gray whale feeding depth, which we set at −75 m. Although most of the current benthic resources of the Bering Sea are located a depths much shallower than the mean depth of the shelf (<−50 m), gray whale feeding pits along so-called tertiary feeding areas off the coast of California and Oregon have been noted at depths of −75 m. We therefore sought to use this latter value, which provided a generous margin for estimating the possible range of feeding benthos in the past.

Besides uniformitarian assumptions about feeding ecology, there are also other alternative hypotheses about processes that have limited gray whale carrying capacity in the geologic past. One consideration could be intrinsic factors, such as changes in fecundity (e.g., number of calves, birth rate), although these specific factors are not testable with current datasets. Among extrinsic factors, benthos availability was the easiest to quantify and constrain over geologic time, but other extrinsic factors include changes in predation, ice cover and regional productivity. Ignoring humans, killer whales (*Orcinus orca*) are the dominant predators of gray whales, and their selective attacks at key junctures along the gray whale coastal migration route in the eastern North Pacific has demonstrable impacts on gray whale population size and life history parameters [50]. Unfortunately, the Pleistocene fossil record of *Orcinus* is negligible, especially in the North Pacific, which severely limits the testability of such an interaction in the late Pleistocene. We discuss the two other extrinsic factors in the following section.

### Impact of ice cover and differential productivity

Large regions of the Bering Sea, the Sea of Okhotsk, and the northern Pacific Ocean experienced extensive seasonal ice sheet cover during glacial periods from the Pleistocene to the beginning of the Holocene [51]–[54]. During glacial periods, ice floes moved across the southern Bering Sea and entered coastal areas of the eastern North Pacific Ocean to create a cold, low salinity surface layer that prevented vertical mixing and lowered regional productivity [51]. The presence of coastal and island glaciers, and fast, anchor, and pack ice undoubtedly further reduced the remaining gray whale feeding habitat in the Bering Sea that was not lost to sea-level drops during glacial maxima. Glaciation would have also produced significant ice-rafted debris, present in along the eastern North Pacific margin as well as in the Sea of Okhotsk [55] and icebergs from calving glaciers would have scoured the nearshore benthos. In the Antarctic, sea ice, anchor ice, and grounded icebergs significantly alter benthic community structure [56]–[59], and it is parsimonious to assume similarly for North Pacific Pleistocene settings. Our analyses did not explicitly account for how such alteration would affect benthos productivity, but we instead simplified our comparisons by reconstructing carrying capacity for non-glaciated regions.

**Figure 4:**
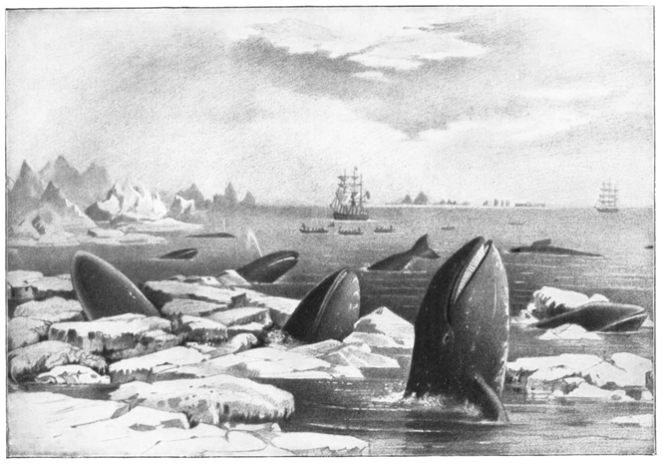
Gray whales amidst ice in the eastern North Pacific Ocean. Taken as anecdotal evidence, this illustration, reproduced from [25], provides insight into gray whale behavioral plasticity, especially in the presence of sea and pack ice. Several observations (e.g., [59]) suggest that gray whales possess a latent ability to tolerate ice, which would be a beneficial trait during episodes of glacial maxima.

Gray whales appear to have a variable capacity to deal with ice. Berzin [60] suggested that their northern distributions were limited by pack ice, and Moore et al. [36] reported that gray whales were almost seven times more likely to be associated with open water and light ice versus habitats with >20% ice cover. Notwithstanding, Scammon ([25], **Figure 4**) described and illustrated gray whales in pack ice. More recently, Stafford et al. [61] recently documented evidence for gray whales overwintering in the Beaufort Sea. Given this range in ice toleration, we suspect that our reconstructed carrying capacities may be overestimates because they account for sea-level-mediated effects of glaciations on feeding habitat, and not the impacts associated with increased sea and continental ice cover. In other words, all of our initial estimates assumed gray whales were feeding directly adjacent to continental and island margins that were free of glaciers, as well as pack and fast sea ice. Instead, if we assumed that gray whales typically avoided glacial and pack ice conditions, carrying capacity estimates were substantial reduced, especially in the eastern North Pacific (**Figure 3B,D**). Moreover, most of carrying capacity at glacial maxima would have been limited to the western North Pacific region (i.e., WPS), and the eastern North Pacific carrying capacity would not have exceeded 1650 individuals during LGM (**Figure 3D** **, Table S2**).

In addition to ice cover, productivity regimes after LGM would have differed from today's values in the North Pacific Ocean because benthic productivity would have been affected by decreased salinity from freshwater runoff and increased sedimentation rates and turbidity from glacial sediments [62]–[64]. Such patterns are evident today from freshwater river systems that impact the nearshore benthos especially along the coasts of the Aleutian Islands, southeast Alaska and British Columbia. Thus, benthic productivity in the Bering and Chukchi seas is 3–4 times higher than coastal environments in Alaska [34]. As with ice cover, accounting for such a consideration would likely lower our estimated carrying capacities further.

### Additional gray whale populations and genetic bottlenecks

We constrained our analyses to postdate the last possible connection between the Pacific and Atlantic ocean basins, which were likely confluent ∼120 ka (late Sangamonian) [16]. Such a connection would have provided a potential dispersal route for gray whales between the Atlantic and the Pacific oceans, and possible increased population size (but not eastern Pacific carrying capacity) to an uncertain degree. Nonetheless, an extensive Holocene record of gray whale skeletal elements along the margin of the North Atlantic Ocean, extending from the southeastern United States through Iceland, the United Kingdom and to Europe [17]–[19] provides ample evidence for the geographic expanse of this population, which likely went extinct within historical times [17]. Interestingly, the documented occurrences of a gray whale off the coast of Israel and Spain in 2010 suggests that prey resources and long-range dispersal do not appear to be barriers preventing future recolonization of this basin, especially during interglacial conditions [65]. A recent discovery of Pleistocene age gray whale material from the coast of Georgia, U.S.A., demonstrates the antiquity of the Atlantic presence of this lineage [66], although not necessarily its continuity. As Alter et al. [16] indicated, it is possible that Atlantic gray whales populations contributed to the genetic diversity of Pacific gray whale populations during interglacial periods, thereby increasing the population size (as well as genetic diversity) in the North Pacific Ocean. Ultimately, we argue that the impact of such a contribution is minimized by restricting our analyses to a time frame postdating 120 ka.

Using the 22 k constraint, it is clear that the absence of the Bering shelf would have pushed carrying capacities to very low levels (**Figure 3C,D**). The repeated loss of the shelf might have only produced weak bottlenecks; given that the lowest carrying capacity estimates ranged in thousands of individuals, we would only expect to recover signals of much stronger genetic bottlenecks from inbreeding between tens of individuals [67], [68]. Using coalescence simulations, Alter et al. [15] found that a genetic bottleneck before 1,100–1,600 years ago was statistically improbable and that any substantial population decline was more likely associated with whaling than any deeper signal. Alter and Rosenbaum [69] reached a similar conclusion, but also reported a possible 20% population decline approximately 600–700 years ago based on ancient DNA recovered from midden material from Washington State. Although unrelated to Pleistocene glaciation events, the timing of this Holocene decline is coincident with the start of the Little Ice Age and increasing ice cover across the Northern Hemisphere for a brief interval. This latter result also provides supporting evidence for the impact of ice cover on gray whale population size and further argues that the 22 k carrying capacity constraint is unlikely in the past, given today's genetic diversity. Benthos availability in the western North Pacific (**Figure 3**) also suggests that the western North Pacific region may have served as a reservoir for genetic diversity during glaciations (**Figure 3C,D**); an intriguing result given that the current population of this region numbers in the hundreds, compared to >20 k gray whales currently alive in the eastern Pacific Ocean. However, the long-range movement of a tagged gray whale from the western to eastern margins of the North Pacific in 2010–2011 hints that the separation between these two populations may be fluidic [70].

If we consider only benthos availability, then there is no incongruity between our results and those of Alter et al. [15]. However, if taking glacial extent and the effects of pack and continental ice on gray whale behavior and feeding habitat into consideration, the harmonic mean of our estimated carrying capacity was below their minimum population size of 76 k individuals. Moreover, as outlined above, the gradual buildup of continental and island glaciers over 95 ka would have resulted in a gradual increase in ice effects, as glacier and pack ice expanded southward. Thus, we suspect that carrying capacity estimates of the glaciated regions would have fallen over time, rather than exhibiting a binary availability because of ice effects. Consequently, the harmonic mean for population was higher than the 34,308 individuals estimated in **Figure 3C**.

Because Earth history constrains the generation of more benthos, any additional feeding habitat to support a larger population would arise only from behavioral changes to gray whale feeding ecology. We therefore suggest that gray whales survived glacial episodes and maintained larger population sizes than benthos availability would support by employing a diverse set of feeding modes, similar to those seen in resident sub-populations along the coastal eastern North Pacific areas of Washington State and Vancouver Island today (i.e., British Columbia and Pacific Northwest of **Figure S1**).

### The case for ecological plasticity in gray whales

**Figure 5:**
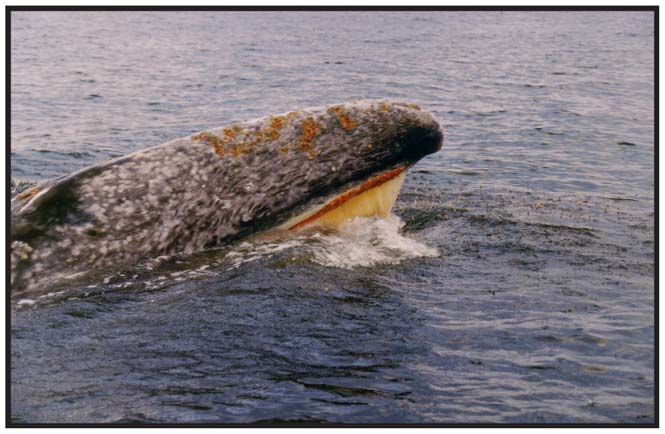
Non-migratory gray whale feeding. Photograph of a gray whale feeding on herring near Cape Scott, Vancouver Island, British Columbia, 17 April 2000. Photograph and observations by the late Donovan Girard, courtesy of K. Lihou and R. Graham.

Although their abilities as benthic feeders dominate many descriptions of their feeding ecology [23]–[27], [37], gray whales also feed on other prey using a more generalized mode of filter-feeding (**Figure 5**), despite the morphological specializations of their baleen, mandibles and throat for suction feeding [27], [71]. In the Bering region, gray whales feed predominately on benthic tube-dwelling amphipods and polychaete worms, but they also have the capability to feed on demersal and pelagic prey items, including a wide range of crustaceans (both adults and larval stages), bony fish (adults, eggs and larvae) and cephalopods [22], [72]–[74]. Most of this feeding variability occurs outside of the Bering region in seasonal resident gray whale populations of the Pacific Northwest and along their migratory route. Resident populations near Washington State and Vancouver Island were first documented in the 1960s and 1970s [75], [76]; by 1998, these seasonal residents numbered over 155 individuals or approximately 1% of the eastern gray whale population [48]. It is uncertain if any genetic structure exists in this seasonal resident gray whale ecotype, but their diminutive numbers, relative to the better-known migratory gray whales likely explain the discrepancy of this ecological mode described in the literature.

We propose that feeding plasticity in gray whales, during the Pleistocene, would have been maximized during eustatic lowstands leading up to and including glacial maxima, when the extensive benthic feeding areas in the Bering, Chukchi and Beaufort seas were eliminated ([37]: 306). The loss of this feeding area was not a one-time event, where a fortuitous behavioral adaptation permitted their survival. Rather, these large-scale habitat changes during glacial maxima, with concomitant sea-level changes, which occurred more than 40 times within the stratigraphic range of the genus *Eschrichtius* (**Figure 1**). Such ecotypic plasticity is not unusual in the comparison with other populations that are predominantly migratory, and examples include bony fish from New Zealand and artiodactyls from the Holarctic and Africa [77]–[79]. Such plasticity buffers populations from sudden and major shifts to food availability, and we propose that the ecological breadth of gray whale feeding modes allowed them to take advantage of alternative food resources and/or feeding areas during glacial maxima. It is unclear whether this plasticity is pleisomorphic (ancestral) or if it evolved in the context of abundant pelagic, demersal, and benthic prey species that gray whales encountered over longer migrations during interglacial periods.

During glacial periods, gray whale migratory routes were likely to have been substantially contracted, with the northern extent of their migration delimited by the margin of the continental ice sheets and the southern limit imposed by higher water temperatures associated with tropical latitudinal gradients, which did not shift southward during glacial maxima [80], [81]. Also, a thermal limitation on the southern extent of gray whale distribution may be inferred by their reduced numbers in the southernmost part of their range during El Niño events when water temperatures rise by 4.2–5.8°C [82].

We envision that Pleistocene gray whale feeding areas were broadly distributed along the eastern and western North Pacific margins similar to those of the current seasonal resident gray whales (sensu [48]) of coastal British Columbia and Washington State, and extended even further south during glacial maxima. During the onset of these glacial episodes, migration distances between different feeding and breeding areas would likely have substantially varied as well because of appearance of different benthic feeding areas and the disappearance of estuaries in Baja California associated with sea-level change (see also [83]). If Pleistocene gray whale populations exhibited the broad ecotypes hypothesized here, why, then, are the ecological distinctions between non-migratory and migratory gray whales so disproportionate? Available historical explanations, such as killer whale (*Orcinus*) avoidance [50] or hyperabundance of non-benthic prey species, are not temporally specific and are not supported by known historical data. We argue below that the preponderance of evidence points to past human disturbances of more southern resident populations, which explain the recent appearance of summer feeding populations outside of the Bering Sea, as well as selecting for the recent, stereotypic, long-distance migration that characterizes this species.

### Human impacts and the post-LGM world

The first humans entered North America from Asia as the last glacial maximum began to subside between 30–16 ka ago [84], already possessing sophisticated abilities to hunt in and collect from nearshore marine habitats [85]. As they dispersed through Alaska and along coastal habitats, they exploited abundant marine mammal assemblages tied to coastal ecosystems along the southern margin of the retreating ice sheets, as they had done previously in Asia. The subsequent effects of these human activities on North Pacific marine mammal populations during the Holocene are especially well documented [86], [87], including widespread extirpations of Northern fur seals (*Callorhinus ursinus*) [10], [88], sea otters (*Enhydra lutra*) [89], Northern elephant seals (*Mirounga angustirostris*) [90]. Human hunting also likely exacerbated the decline of Steller's sea cow (*Hydrodamalis gigas*), culminating with its extinction during historical times [5].

Gray whales would have been similarly affected by arriving humans, whose shore-based whaling technologies along coastal areas would have been sufficient, without the need for more pelagic modes of hunting [91]–[94]. Resident whale populations are likely to have been the most vulnerable because of key ecological characteristics: their close proximity to the coast; their preference for sheltered embayments, straits and passages; and their long local residence time. We would expect that resident populations were first extirpated, and that whatever resident populations remained at the time of European contact were quickly extirpated as well. As shore-based whaling became inefficient, ship-based whaling made possible the systematic reduction of the remaining migratory population at their feeding grounds in the Bering, Chukchi and Beaufort Seas and their calving grounds in Baja California, Mexico [29]. We cite the temporal co-occurrence of seasonal resident gray whales appearances and the suspension of human hunting as support for the argument that subsequent, post-whaling population growth in the late 20^th^ century [48] permitted the manifestation of the feeding plasticity that resides within the gray whale lineage. Moreover, the secondary morphological specializations that gray whales possess for benthic suction feeding do not impede a more plesiomorphic mode of filter feeding in the water column (**Figure 4**), an approach adopted by seasonal residents. We argue here that the maintenance of this ancestral condition, rather than derived features related to benthic suction feeding, provided the ecomorphologic apparatus to survive an interval of regular and rapid sea-level change during the glaciation of the Northern Hemisphere (**Figure 2**).

### Implications for conservation

We suggest that any extensions or special provisions for protecting gray whales should explicitly favor resident gray whales in the coastal areas of the eastern North Pacific (i.e., British Columbia and Washington State) because they exhibit an important behavioral plasticity that confers an increased fitness for the entire population in the North Pacific Ocean. It is unclear if resident gray whales are genetically distinct from other gray whales, but we suggest that such ecological plasticity in feeding will be an important trait with the increasingly rapid heating of the Northern cryosphere projected to occur in the coming decades [95]. Beyond benthic availability, there are additional causes that may restrict gray whale population size to its current level, given the known changes to the Bering Sea ecosystem [34], which may have altered the capacity of nearshore foodwebs to support such important habitat modifying predators. Nonetheless, protecting those individuals that display alternative migratory behavior and feeding modes should be an important priority regardless of their molecular or morphological similarity [96].

### Conclusion

Marine ecologists have become increasingly aware that the fundamental baselines used to measure species diversity and distributions have shifted in the course of human history [97]–[99]. For marine mammals, many population sizes have been heavily diminished by sustained human hunting, in different parts of the world and at varying levels of intensity, for over 1000 years [12]. For gray whales, which teetered close to extinction, their recovery in the eastern North Pacific Ocean has renewed questions about baselines for their population size, prior to whaling, and subsequently has produced conflicting estimates from different datasets [16], [49]. Our effort here attempted to understand the differences in these baselines at the scale of geologic time, based on known constraints from gray whale feeding ecology (e.g., benthos availability, diving depth). In effect, our study used Earth and ocean history in the late Pleistocene to set boundary conditions and test these competing population estimates by examining feeding areas through time and adjusting whale biomass relative to habitat availability. Previous authors have suggested that glacial maxima affected the distribution of gray whales [e.g.], [ 35, 37], but these suggestions fell short of quantifying estimates of potential population size through a glacial-interglacial cycle.

Our results demonstrated that gray whales in the North Pacific Ocean would have undergone substantial population fluctuations if they were constrained to suction feeding on benthic prey items. Although it is not possible to currently resolve the presence or absence of such deep genetic signals, we hypothesized that gray whales survived the Pleistocene because of a greater range of feeding modes and a less canalized migratory behavior that allowed for them to feed outside of the Sea of Okhotsk and Bering region and away from ice cover and glaciers. Sequential human occupation around the rim of the North Pacific exploited and reduced these populations and commercial whaling finally expatriated them. However, the recent observations of seasonally resident populations along the coast from northern California to southeastern Alaska suggests that plasticity in feeding mode is still present in the lineage. We proposed that with continued human diligence, gray whales might return to a more typical interglacial distribution and abundance. Lastly, our modeling of benthic habitat through time provides an important context for understanding the history of other important ecologically important consumers and predators in the Bering Sea ecosystem (e.g., sea otters, walruses, eider ducks), which, like gray whales, have also survived many glacial episodes when the Chukchi, Beaufort, and Bering shelf communities mostly disappeared. To address impending biological changes to these marine ecosystems, we underscore the importance of integrating data from earth and ocean history, in the context of known ecological traits and values.

## Materials and Methods

### Fossil record of gray whales

The holotype of *E. robustus* is based on a sub-fossil (i.e., Holocene) specimen from marine sand and clay deposits in Gräsö, Sweden [100]. It is likely that this specimen belonged to the now extinct population of Atlantic gray whales, which are represented by numerous Holocene localities along both western and eastern coastlines of the North Atlantic Ocean [17], [19]. The pre-Holocene fossil record of Eschrichtiidae is otherwise sparse, compared with the abundance of other fossil mysticetes, and the Pleistocene record of most marine mammals is globally poor [44]. The sole Pleistocene record assigned to the species of *E. robustus* in the North Pacific is a partial skeleton and a nearly complete, associated skull recovered from the San Pedro Sand of Los Angeles County, California [101]. In their original description, Barnes and McLeod [101] indicated that the San Pedro Sand was 125 ka. Recent revision of the age of that rock unit, however, places the age of this specimen between ∼500–200 ka [102], although it is likely closer to the younger estimate of 200 ka (T. A. Deméré, pers. comm.). A much older fossil gray whale, referred only to the generic level (*Eschrichtius* sp.) was reported from late Pliocene rocks on the island of Hokkaido, Japan [103], further extending the age of this lineage to ∼2.6–3.9 Ma.

Although these occurrences reliably extend the record of *E. robustus* well past the last glacial-interglacial cycle to at least 200 ka (and extend the presence *Eschrichtius* in the North Pacific basin to at least the late Pliocene), fossil remains of close gray whale relatives, within Eschrichtiidae, broaden geographic extent of this group. In the North Pacific Ocean, Deméré et al. [104] reported an undescribed and unnamed eschrichtiid species from the San Diego Formation, of similar age to the late Pliocene *Eschrichtius* sp. occurrence in Japan, which appears to be the sister taxon to *Eschrichtius*. Whitmore and Kaltenbach [105] named *Gricetoides aurorae* on the basis of a partial cranium (including a periotic) and referred isolated material from the early Pliocene Yorktown Formation of North Carolina. In the Mediterranean Basin, two additional eschrichtiids have also been recently named. Based on an unusual partial mandible with several diagnostic eschrichtiid features, Bisconti and Varola [106] emended the diagnosis of Eschrichtiidae to classify this specimen as an eschrichtiid, *Archaeschrichtius ruggeroi*, from the late Miocene Pietra Leccese Formation of Italy. Bisconti [107] described another eschrichtiid from Italy, *Eschrichtioides gastaldii*. Consisting of an incomplete skull, mandibles and incomplete postcrania from the early Pliocene Sabbie d'Asti Formation, this other specimen was originally described as *Balaenoptera gastaldii* [104] although key cranial and mandibular features diagnose its placement within Eschrichtiidae. Overall, this Mio-Pliocene record broads the geographic distribution of the clade to include all large oceanic basins in the Northern Hemisphere, and push the antiquity of Eschrichtiidae to the late Miocene. For the purposes of this study, however, we used the aforementioned genus and species level gray whale records from Hokkaido and California, respectively, to constrain our analyses within the North Pacific basin.

### Sea-level record

Several different sea-level curves were available for this study [e.g.], [ 43], [ 108]–[111]. We selected that of Miller et al. [43], because it provided us with a uniform and practical time interval for our study (5 kyr) and it had complete coverage through the period of interest. Previous studies [108], [109] estimated sea level at much larger intervals (e.g., 1 Myr), which did not allow us to resolve the last glacial-interglacial cycle. More recent studies have estimated sea level at smaller time intervals during the last glacial cycle, but the intervals were highly variable (e.g., 0.36–6285 years; m = 181.52 years, s.d. = 334.32) [111] or they lacked estimates at critical intervals during the cycle [110]. However, there is general agreement between Miller et al. [43] and these more recent studies, particularly in regard to the low stand sea-level estimates, which is our focus in this study because they affected gray whale feeding areas in the Bering Sea. The major difference between these studies is the 120 ka high stand value that Miller et al. [43] estimated at +24 m above present sea level; Rohling et al. [111] regarded this value as spurious because it shows a distinct, temporally limited offset that has not been replicated in other cores. Removing this contentious data point from our estimates of gray whale carrying capacity reduces the harmonic means of our unconstrained and 22 k estimates by only 1.7%. Moreover, because such a correction increases feeding area rather than decreasing it, we view this issue as having little effect on our overall results or conclusions.

However, there are numerous other factors that can complicate estimates of past sea-levels. Global sea-level (eustasy) changes reflect long-term (i.e., geologic scale) changes in water volume within large ocean basins. Although many factors (e.g., thermal shifts, freshwater input) can influence the rate and amplitude of sea-level changes, episodic glaciation is mainly responsible for rapid sea-level changes at high amplitudes. For the late Pliocene-Holocene (∼2.5–0 Ma), Miller et al. [43] used a benthic foraminiferal ^18^O record that scaled with sea-level curves from older records (>7 Ma), which instead required stratigraphic backstripping to control for sediment compaction, loading and subsidence. Sea-level curves from ∼2.5 Ma to the present exhibit notable “sawtooth” patterns of gradual ice buildup (i.e., the onset of Northern Hemisphere ice sheets) followed by sudden terminations that have been linked to overlying patterns directed by 10^4^– to 10^5^-year scale Milankovitch cycles [112], [113]. The temporal frame of the sea-level curves used here were bracketed by the youngest stratigraphic occurrence of diagnostic *E. robustus* fossils (0.2 Ma), a boundary that also minimized the effects of tectonism, which is active in along the northern continental margins of the Pacific Ocean. Nonetheless, Miller et al. [43] still cautioned that glacial rebound from isostasy could induce a ±10 m error estimate for sea-level estimates in shallow shelf environments. Because our carrying capacity estimates are based on the maximum feeding depth of the gray whales rather than mean foraging depths (see below), this potential source of error does not dramatically change our estimates of carrying capacity.

### Benthos availability and foraging depths

We divided the cumulative ranges of the North Pacific gray whales populations into 4 regions that divided the continental margins of the eastern and western North Pacific Ocean into areas that were glaciated and non-glaciated during the last 120 ka (**Table 1** **, Figure S1**). Latitude, longitude, and depth or elevation, centered on the intersection of each odd minute, were downloaded from the ETOPO1 database for each region [114]. The ETOPO1 database is a 1 arc-minute global relief model of Earth's surface (including ocean bathymetry), which was built from numerous global and regional data sets. We then calculated the area (km^2^) of each minute by minute cell using the following equation to correct for longitudinal convergence at the poles [115].
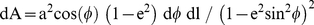
(1)where:

dA = cell area (km^2^)
latitude (*φ*) = latitude of cell's center (in radians)
unit of Latitude (d*φ*) = 1 arc-minute (2.908882×10^−4^ radians)
unit of Longitude (dl) = 1 arc-minute (2.908882×10^−4^ radians)
equatorial radius (a) = 6378.137 km
eccentricity (e) = 0.08181919

Only individual cell areas for depths or elevations between +24 m and −197 m were retained (**Figure S2**). The +24 m value corresponded to sea level at the last interglacial high stand, while the −197 m value represented sea level at the most recent low stand (−122 m) plus −75 m, the assumed maximum feeding depth of gray whales. We then calculated benthic area within 75 m of sea level at 5 ka intervals along the Pleistocene sea level curve of Miller et al. ([43]: table S1) (**Figure 2** **; Table S1**). For example, at 50 ka, all cells between −75 and −150 m were summed for each region providing an estimate of benthos availability at that time (**Figure S2**).

We also assumed all feeding areas within 75 m of the surface were covered by sediment and therefore could support food patches similar to the Bering shelf. This assumption, however, is likely an overestimation because sedimentary benthos occupies only about 65% of the northeastern Pacific coast (J.A. Estes, pers. comm.), thus potentially reducing both our unconstrained and 22 k carrying capacity estimates by as much as 35%. In addition, our use of a 75 m window for feeding depth [46] is substantially greater than the 5–35 m average foraging depth for the western population [22] and the <60 m depth for most of the feeding areas within the Bering and Chukchi Seas (**Figure 2A**). Again, we likely over-estimated the size of past carrying capacities by providing generous mean performance values for foraging.

### Reconstructing past carrying capacities

To reconstruct estimates of gray whale carrying capacity change during the most recent glacial cycle we multiplied available benthos calculated above by food patch density and prey density and then divided this value by gray whale biomass requirements to estimate the maximum number of gray whales that the benthos could support (**Table S2**). To estimate gray whale carrying capacity we used gray whale biomass requirements from Alter et al. [15], feeding window duration from Johnson and Nelson [47], and prey density and food patch size from Nerini [26] (**Table 2**). To limit the carrying capacity of the North Pacific to the current population size (see [33]), we scaled the ratio of food patch size to the benthic area value so that it would support only 22 k individuals (**Table 2**).

## Supporting Information

Figure S1 **Map of the North Pacific Ocean and geographic subdivisions used to generate discrete regions of benthos availability.** See **Table 1** for definitions.
(TIF)

Figure S2 **Benthic sampling profile of depth ranges within 75 m diving depth of gray whales during the last 120 ka.**
(TIF)

Table S1 **Estimated benthos availability (km^2^) within 75 m of the surface during the last 120 ka.** See Materials and Methods for data source and treatment.
(DOC)

Table S2 **Estimated grey whale population sizes in the North Pacific during the last 120 ka under two carrying capacity assumptions.** Regions (**Figure S1**): EPS = eastern Pacific south (non-glaciated); EPN = eastern Pacific North (glaciated); WPS = western Pacific south (non-glaciated); WPN = western Pacific North (glaciated); NP = North Pacific (all 4 regions). Assumptions (see **Table 2**): UC = unconstrained carrying capacity; 22 K = alternative 22 k gray whale carrying capacity based on current eastern Pacific population size [33].
(DOC)
