# Supplementary figures and images for: What Happened to Gray Whales during the Pleistocene? The Ecological Impact of Sea-Level Change on Benthic Feeding Areas in the North Pacific Ocean
Source: PLoS One. 2011 Jul 6;6(7):e21295. doi: 10.1371/journal.pone.0021295 (PMC3130736; doi:10.1371/journal.pone.0021295)

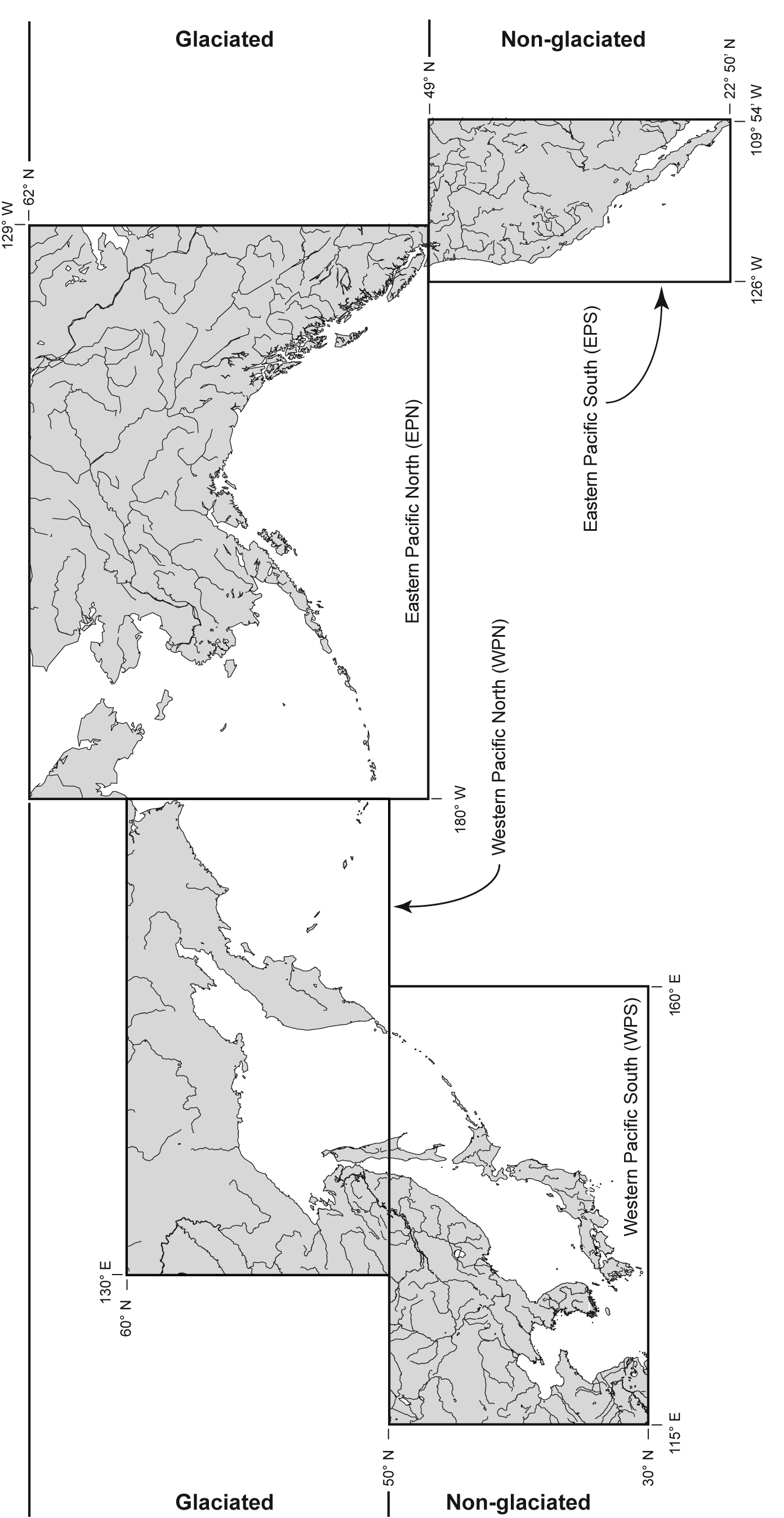

Supplement: Figure S1 — Map of the North Pacific Ocean and geographic subdivisions used to generate discrete regions of benthos availability. See Table 1 for definitions. (TIF) [file pone.0021295.s001.tif]

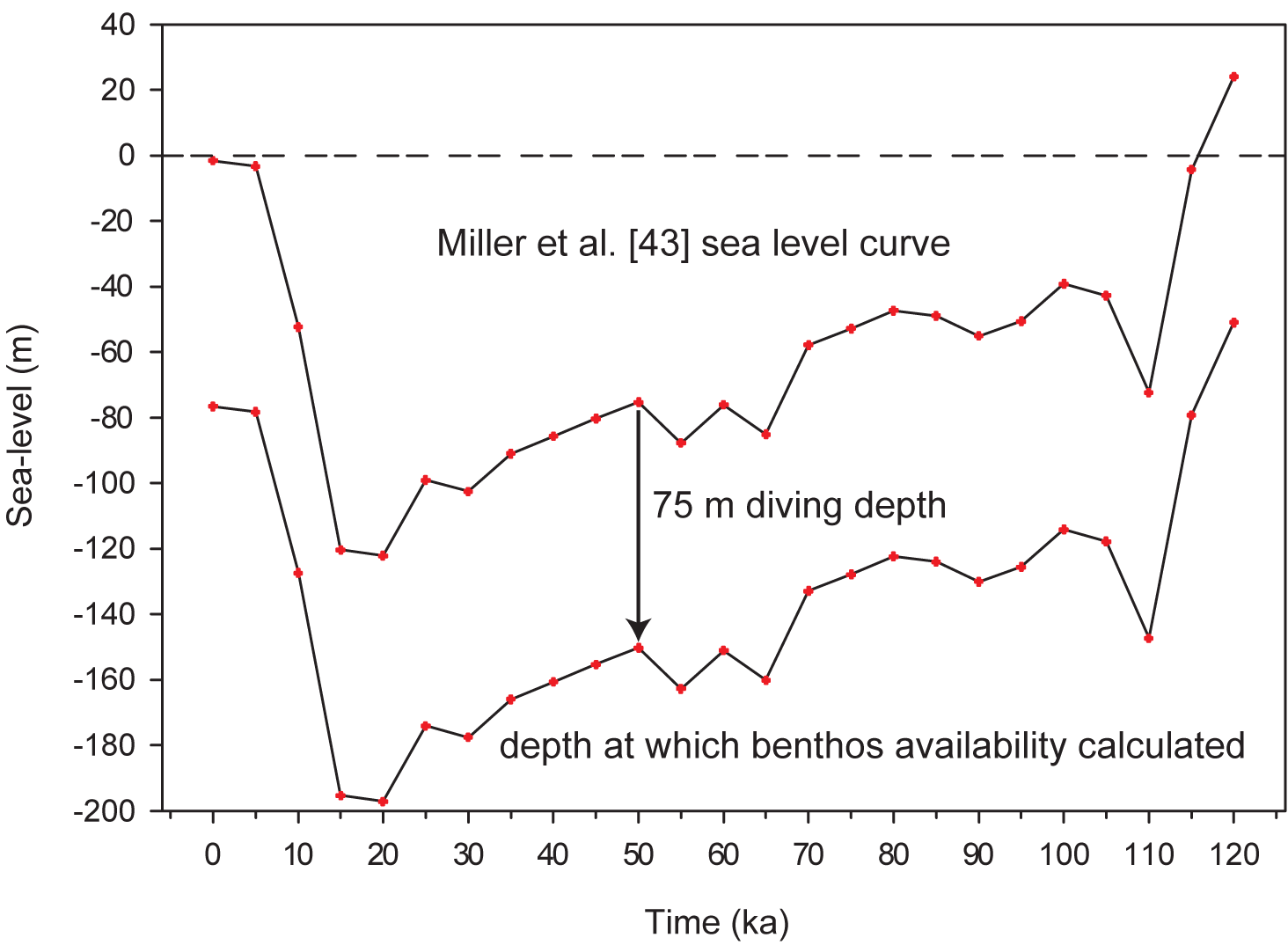

Supplement: Figure S2 — Benthic sampling profile of depth ranges within 75 m diving depth of gray whales during the last 120 ka. (TIF) [file pone.0021295.s002.tif]
