# Supplementary material for: What Happened to Gray Whales during the Pleistocene? The Ecological Impact of Sea-Level Change on Benthic Feeding Areas in the North Pacific Ocean
Source: PLoS One. 2011 Jul 6;6(7):e21295. doi: 10.1371/journal.pone.0021295 (PMC3130736; doi:10.1371/journal.pone.0021295)
Supplement: Table S1 — Estimated benthos availability (km2) within 75 m of the surface during the last 120 ka. See Materials and Methods for data source and treatment. (DOC) [file pone.0021295.s003.doc]

TABLE S1.

| |  |  | **Benthos ≤ 75 m** | | | | | | | --- | --- | --- | --- | --- | --- | --- | --- | |  | **Sea level** | **EPS** | **EPN** | **WPN** | **WPS** | **NP** |  | | **ka** | **m** | **km^2^** | | | | | | | 0 | -1.6 | 56271 | 867193 | 226031 | 733964 | 1883459 |  | | 5 | -3.3 | 55195 | 861567 | 224846 | 731276 | 1872883 |  | | 10 | -52.4 | 63653 | 564675 | 198554 | 452791 | 1279673 |  | | 15 | -120.4 | 29341 | 223680 | 215358 | 164897 | 633276 |  | | 20 | -122.2 | 28445 | 216875 | 212160 | 160806 | 618286 |  | | 25 | -99.1 | 42656 | 302847 | 245269 | 227177 | 817949 |  | | 30 | -102.6 | 39722 | 286713 | 241418 | 210668 | 778521 |  | | 35 | -91.0 | 48473 | 342870 | 249136 | 253177 | 893655 |  | | 40 | -85.7 | 52200 | 370964 | 249162 | 272427 | 944753 |  | | 45 | -80.4 | 55610 | 408031 | 247585 | 303713 | 1014939 |  | | 50 | -75.3 | 58249 | 443573 | 243658 | 331364 | 1076844 |  | | 55 | -87.8 | 50677 | 359223 | 249383 | 264333 | 923617 |  | | 60 | -76.1 | 57849 | 436048 | 244665 | 325620 | 1064182 |  | | 65 | -85.2 | 52778 | 376340 | 249150 | 276865 | 955133 |  | | 70 | -57.9 | 63617 | 528256 | 214012 | 426010 | 1231895 |  | | 75 | -52.9 | 63803 | 557369 | 201276 | 448811 | 1271258 |  | | 80 | -47.4 | 64379 | 641803 | 196482 | 494738 | 1397401 |  | | 85 | -49.0 | 63885 | 607985 | 195096 | 475332 | 1342299 |  | | 90 | -55.1 | 63843 | 545881 | 206332 | 439871 | 1255927 |  | | 95 | -50.6 | 63573 | 573711 | 196428 | 456378 | 1290090 |  | | 100 | -39.2 | 64608 | 722591 | 200804 | 535174 | 1523177 |  | | 105 | -42.8 | 64771 | 688370 | 198880 | 518922 | 1470942 |  | | 110 | -72.4 | 59482 | 465261 | 240270 | 352320 | 1117333 |  | | 115 | -4.3 | 54975 | 858255 | 222839 | 728213 | 1864282 |  | | 120 | 24.0 | 58971 | 895340 | 228590 | 732761 | 1915662 |  | |  |  |  |  |  |  |  |  |  |
| --- | --- | --- | --- | --- | --- | --- | --- | --- | --- | --- | --- | --- | --- | --- | --- | --- | --- | --- | --- | --- | --- | --- | --- | --- | --- | --- | --- | --- | --- | --- | --- | --- | --- | --- | --- | --- | --- | --- | --- | --- | --- | --- | --- | --- | --- | --- | --- | --- | --- | --- | --- | --- | --- | --- | --- | --- | --- | --- | --- | --- | --- | --- | --- | --- | --- | --- | --- | --- | --- | --- | --- | --- | --- | --- | --- | --- | --- | --- | --- | --- | --- | --- | --- | --- | --- | --- | --- | --- | --- | --- | --- | --- | --- | --- | --- | --- | --- | --- | --- | --- | --- | --- | --- | --- | --- | --- | --- | --- | --- | --- | --- | --- | --- | --- | --- | --- | --- | --- | --- | --- | --- | --- | --- | --- | --- | --- | --- | --- | --- | --- | --- | --- | --- | --- | --- | --- | --- | --- | --- | --- | --- | --- | --- | --- | --- | --- | --- | --- | --- | --- | --- | --- | --- | --- | --- | --- | --- | --- | --- | --- | --- | --- | --- | --- | --- | --- | --- | --- | --- | --- | --- | --- | --- | --- | --- | --- | --- | --- | --- | --- | --- | --- | --- | --- | --- | --- | --- | --- | --- | --- | --- | --- | --- | --- | --- | --- | --- | --- | --- | --- | --- | --- | --- | --- | --- | --- | --- | --- | --- | --- | --- | --- | --- | --- | --- | --- | --- | --- | --- | --- | --- | --- | --- | --- | --- | --- | --- | --- | --- | --- | --- | --- | --- |
|  |  |  |  |  |  |  |  |  |  |
