# Supplementary material for: What Happened to Gray Whales during the Pleistocene? The Ecological Impact of Sea-Level Change on Benthic Feeding Areas in the North Pacific Ocean
Source: PLoS One. 2011 Jul 6;6(7):e21295. doi: 10.1371/journal.pone.0021295 (PMC3130736; doi:10.1371/journal.pone.0021295)
Supplement: Table S2 — Estimated grey whale population sizes in the North Pacific during the last 120 ka under two carrying capacity assumptions. Regions (Figure S1): EPS = eastern Pacific south (non-glaciated); EPN = eastern Pacific North (glaciated); WPS = western Pacific south (non-glaciated); WPN = western Pacific North (glaciated); NP = North Pacific (all 4 regions). Assumptions (see Table 2 ): UC = unconstrained carrying capacity; 22 K = alternative 22 k gray whale carrying capacity based on current eastern Pacific population size [33]. (DOC) [file pone.0021295.s004.doc]

\

| **TABLE S2.­**   | Time | Sea level | Regional population estimates with unconstrained (UC) and 22K carrying capacities | | | | | | | | | | | --- | --- | --- | --- | --- | --- | --- | --- | --- | --- | --- | --- | | ka | m | EPS UC | EPS 22K | EPN UC | EPN 22K | WPN UC | WPN 22K | WPS UC | WPS 22K | NP UC | NP 22K | | 0 | -1.6 | 5110 | 1428 | 78290 | 22006 | 20406 | 5736 | 66262 | 18625 | 170039 | 47795 | | 5 | -3.3 | 4983 | 1401 | 77782 | 21863 | 20299 | 5706 | 66020 | 18557 | 169084 | 47526 | | 10 | -52.4 | 5747 | 1615 | 50979 | 14329 | 17925 | 5039 | 40878 | 11490 | 115529 | 32473 | | 15 | -120.4 | 2649 | 745 | 20194 | 5676 | 19443 | 5465 | 14887 | 4184 | 57172 | 16070 | | 20 | -122.2 | 2568 | 722 | 19579 | 5503 | 19154 | 5384 | 14518 | 4081 | 55819 | 15690 | | 25 | -99.1 | 3851 | 1082 | 27341 | 7685 | 22143 | 6224 | 20510 | 5765 | 73844 | 20756 | | 30 | -102.6 | 3586 | 1008 | 25884 | 7276 | 21795 | 6126 | 19019 | 5346 | 70285 | 19756 | | 35 | -91.0 | 4376 | 1230 | 30954 | 8701 | 22492 | 6322 | 22857 | 6425 | 80679 | 22677 | | 40 | -85.7 | 4713 | 1325 | 33491 | 9414 | 22494 | 6323 | 24595 | 6913 | 85292 | 23974 | | 45 | -80.4 | 5020 | 1411 | 36837 | 10354 | 22352 | 6283 | 27419 | 7707 | 91629 | 25755 | | 50 | -75.3 | 5259 | 1478 | 40046 | 11256 | 21997 | 6183 | 29916 | 8409 | 97217 | 27326 | | 55 | -87.8 | 4575 | 1286 | 32431 | 9116 | 22514 | 6328 | 23864 | 6708 | 83384 | 23438 | | 60 | -76.1 | 5223 | 1468 | 39366 | 11065 | 22088 | 6209 | 29397 | 8263 | 96074 | 27005 | | 65 | -85.2 | 4765 | 1339 | 33976 | 9550 | 22493 | 6322 | 24995 | 7026 | 86229 | 24237 | | 70 | -57.9 | 5743 | 1614 | 47691 | 13405 | 19321 | 5431 | 38460 | 10810 | 111215 | 31261 | | 75 | -52.9 | 5760 | 1619 | 50319 | 14144 | 18171 | 5108 | 40519 | 11389 | 114769 | 32259 | | 80 | -47.4 | 5812 | 1634 | 57942 | 16286 | 17738 | 4986 | 44665 | 12554 | 126157 | 35460 | | 85 | -49.0 | 5768 | 1621 | 54889 | 15428 | 17613 | 4951 | 42913 | 12062 | 121183 | 34062 | | 90 | -55.1 | 5764 | 1620 | 49282 | 13852 | 18628 | 5236 | 39712 | 11162 | 113385 | 31870 | | 95 | -50.6 | 5739 | 1613 | 51795 | 14558 | 17734 | 4985 | 41202 | 11581 | 116469 | 32737 | | 100 | -39.2 | 5833 | 1639 | 65235 | 18336 | 18129 | 5096 | 48315 | 13581 | 137512 | 38652 | | 105 | -42.8 | 5848 | 1644 | 62146 | 17468 | 17955 | 5047 | 46848 | 13168 | 132797 | 37327 | | 110 | -72.4 | 5370 | 1509 | 42004 | 11806 | 21692 | 6097 | 31807 | 8940 | 100873 | 28353 | | 115 | -4.3 | 4963 | 1395 | 77483 | 21779 | 20118 | 5655 | 65743 | 18479 | 168307 | 47308 | | 120 | 24.0 | 5324 | 1496 | 80831 | 22720 | 20637 | 5801 | 66154 | 18595 | 172946 | 48612 |   ­ |  |  |  |  |  |  |  |  |  |
| --- | --- | --- | --- | --- | --- | --- | --- | --- | --- | --- | --- | --- | --- | --- | --- | --- | --- | --- | --- | --- | --- | --- | --- | --- | --- | --- | --- | --- | --- | --- | --- | --- | --- | --- | --- | --- | --- | --- | --- | --- | --- | --- | --- | --- | --- | --- | --- | --- | --- | --- | --- | --- | --- | --- | --- | --- | --- | --- | --- | --- | --- | --- | --- | --- | --- | --- | --- | --- | --- | --- | --- | --- | --- | --- | --- | --- | --- | --- | --- | --- | --- | --- | --- | --- | --- | --- | --- | --- | --- | --- | --- | --- | --- | --- | --- | --- | --- | --- | --- | --- | --- | --- | --- | --- | --- | --- | --- | --- | --- | --- | --- | --- | --- | --- | --- | --- | --- | --- | --- | --- | --- | --- | --- | --- | --- | --- | --- | --- | --- | --- | --- | --- | --- | --- | --- | --- | --- | --- | --- | --- | --- | --- | --- | --- | --- | --- | --- | --- | --- | --- | --- | --- | --- | --- | --- | --- | --- | --- | --- | --- | --- | --- | --- | --- | --- | --- | --- | --- | --- | --- | --- | --- | --- | --- | --- | --- | --- | --- | --- | --- | --- | --- | --- | --- | --- | --- | --- | --- | --- | --- | --- | --- | --- | --- | --- | --- | --- | --- | --- | --- | --- | --- | --- | --- | --- | --- | --- | --- | --- | --- | --- | --- | --- | --- | --- | --- | --- | --- | --- | --- | --- | --- | --- | --- | --- | --- | --- | --- | --- | --- | --- | --- | --- | --- | --- | --- | --- | --- | --- | --- | --- | --- | --- | --- | --- | --- | --- | --- | --- | --- | --- | --- | --- | --- | --- | --- | --- | --- | --- | --- | --- | --- | --- | --- | --- | --- | --- | --- | --- | --- | --- | --- | --- | --- | --- | --- | --- | --- | --- | --- | --- | --- | --- | --- | --- | --- | --- | --- | --- | --- | --- | --- | --- | --- | --- | --- | --- | --- | --- | --- | --- | --- | --- | --- | --- | --- | --- | --- | --- | --- | --- | --- | --- | --- | --- | --- | --- | --- | --- | --- | --- | --- | --- | --- | --- | --- | --- | --- | --- | --- | --- | --- | --- |
